# PEGylation and antioxidant effects of a human glutathione peroxidase 1 mutant

**DOI:** 10.18632/aging.203822

**Published:** 2022-01-12

**Authors:** Guang-Yuan Zhang, Yan-Wei Wang, Li-Ying Guo, Liang-Ru Lin, Shao-Peng Niu, Chang-Hao Xiong, Jing-Yan Wei

**Affiliations:** 1 College of Pharmaceutical Science, Jilin University, Changchun 130021, PR China; 2 Key Laboratory for Molecular Enzymology and Engineering of the Ministry of Education, Jilin University, Changchun 130000, PR China

**Keywords:** PEGylation, human glutathione peroxidase 1 mutant, antioxidant, cardiotoxicity, thermal stability

## Abstract

Human glutathione peroxidase1 (hGPx1) is a good antioxidant and potential drug, but the limited availability and poor stability of hGPx1 have affected its development and application. To solve this problem, we prepared a hGPx1 mutant (GPx1M) with high activity in an *Escherichia coli* BL21(DE3)cys auxotrophic strain using a single protein production (SPP) system. In this study, the GPx1M was conjugated with methoxypolyethylene glycol-succinimidyl succinate (SS-mPEG, *M*_w_ = 5 kDa) chains to enhance its stability. SS-mPEG-GPx1M and GPx1M exhibited similar enzymatic activity and stability toward pH and temperature change, and in a few cases, SS-mPEG-GPx1M was discovered to widen the range of pH stability and increase the temperature stability. Lys 38 was confirmed as PEGylated site by liquid-mass spectrometry. H9c2 cardiomyoblast cells and Sprague-Dawley (SD) rats were used to evaluate the effects of GPx1M and SS-mPEG-GPx1M on preventing or alleviating adriamycin (ADR)-mediated cardiotoxicity, respectively. The results indicated that GPx1M and SS-mPEG-GPx1M had good antioxidant effects *in vitro* and *in vivo*, and the effect of SS-mPEG-GPx1M is more prominent than GPx1M *in vivo*. Thus, PEGylation might be a promising method for the application of GPx1M as an important antioxidant and potential drug.

## INTRODUCTION

Natural human glutathione peroxidase 1 (hGPx1) is an important antioxidant enzyme that can protect the body from oxidative damage by catalyzing the reduction of hydroperoxides using glutathione (GSH) as a reductant [1]. However, the use of hGPx1 is affected by its limited sources and poor stability [2, 3]. To solve this problem, we prepared a high yield and catalytic activity selenium-dependent glutathione peroxidase 1 mutant (GPx1M) by mutating cysteine (C 78, C 115, and C 156) in hGPx1 to serine in an *Escherichia coli* BL21(DE3)cys auxotrophic strain using a single protein production (SPP) system [4]. The GPx1M exhibited high activity, which is 5-fold more than natural bovine liver GPx, and its stability was improved compared with the that of hGPx1. As with many other therapeutic peptides and proteins, GPx1 has a low stability and short plasma half-life [5]. These shortcomings limit the clinical application of GPx1 and its mutant protein.

PEGylation, an effective and well-known strategy to prevent the shortcomings of peptides and proteins, has led to 12 marketed drugs [6]. Such as PEGylated asparaginase (Oncaspar^®^) [7], PEGylated interferon α -2b (PEG-Intron^®^) [8], and PEGylated adenosine deaminase (Adagen^®^) [9]. The modification of proteins by the covalent conjugation of polyethylene glycol (PEG) shows great potential for enhancing enzyme stability [10], preventing effects due to temperature, pH, and proteolytic digestion [11], and improving pharmaceutical properties [12, 13]. Proteins conjugated with PEG not only show reduced immunogenicity and renal clearance [14] but also exhibit improved half-time and stability in the blood [15] by preventing hydrolysis with proteolytic enzymes [16]. The circulation of PEGylated proteins can avoid the necessity of multiple injections to patients. This prompted us to consider whether PEGylation of GPx1M could improve its stability but not affect its activity as an antioxidant enzyme. To date, modification of GPx1M by PEG has never been reported, and the antioxidant effect of GPx1M and the modified product at the cellular level has not been reported in literatures.

Adriamycin (ADR) is a quinone-containing drug for treating malignancy [17] that is limited in clinical use due to its severe cardiotoxicity [18]. The toxicity of ADR is attributed to the generation of large amounts of reactive oxygen species (ROS) and lipid peroxidation in cardiomyocytes [19]. Lipid peroxidation is initiated by free radicals, causing the oxidative destruction of poly-unsaturated fatty acids in cellular membranes. This destruction leads to the generation of malondialdehyde (MDA) [20]. Lactate dehydrogenase (LDH) is an intracellular enzyme for indicating cell membrane damage [21], which can be generated by oxidative stress. Therefore, ADR was selected as a model drug to test the antioxidant capacity of GPx1M and its PEGylation.

In the present study, the GPx1M was modified by methoxypolyethylene glycol succinimidyl succinate (SS-mPEG, *M*_w_ = 5 kDa). The synthesis route of SS-mPEG and SS-mPEG conjugated GPx1M (SS-mPEG-GPx1M) was established. SS-mPEG was confirmed by ^1^H nuclear magnetic resonance (^1^H-NMR) spectrometry. SS-mPEG-GPx1M was characterized in terms of sodium dodecyl sulfate-polyacrylamide gel electrophoresis (SDS-PAGE), iodine staining, and matrix-assisted laser desorption ionization time of flight mass spectrometry (MALDI-TOF-MS). PEGylation site was confirmed by the liquid chromatograph-electrospray ionization-mass spectrometer (LC-ESI-MS). The enzyme stability was evaluated at different temperatures and pH values. Cell viability, lipid peroxidation, cell membrane damage, cellular apoptosis and cellular ROS content were also investigated to reveal the protective effects of SS-mPEG-GPx1M and GPx1M on H9c2 cells. Animal experiments were performed to evaluate the antioxidant effects and pharmacokinetic of SS-mPEG-GPx1M *in vivo*. This study developed a PEGylated GPx1M with SS-mPEG for the first time and demonstrated that PEGylated GPx1M had greater potential antioxidant properties than that of GPx1M.

## RESULTS

### Characterization of SS-mPEG

**Figure 1:**
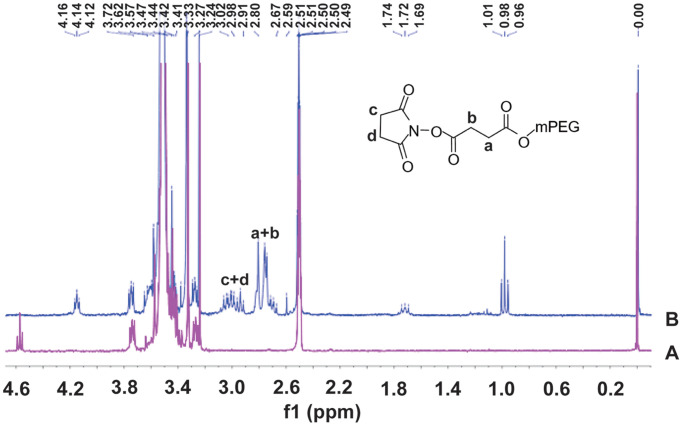
^1^H Nuclear magnetic resonance (^1^H-NMR) spectra of methoxy polyethylene glycol (**A**) and methoxy polyethylene glycol-succinimidyl succinate (**B**) in DMSO_d6_.

The ^1^H-NMR spectra of mPEG and SS-mPEG were shown in Figure 1. Compared to the standard spectrum of mPEG, several new peaks showing a characteristic absorption at δ_2.67–3.24_ ppm were observed for SS-mPEG. The δ_1.69_, δ_1.72_, δ_1.74_ and δ_1.76_ ppm peaks correspond to diethylether methylene protons. The δ_0.96_, δ_0.98_, and δ_1.01_ ppm peaks correspond to diethyl methylene protons. Methylene proton signals from NHS were observed at δ_2.91–3.24_ ppm. The δ_2.67–2.80_ ppm peaks correspond to SA methylene proton signals.

### Preparation and characterization of SS-mPEG-GPx1M

**Figure 2:**
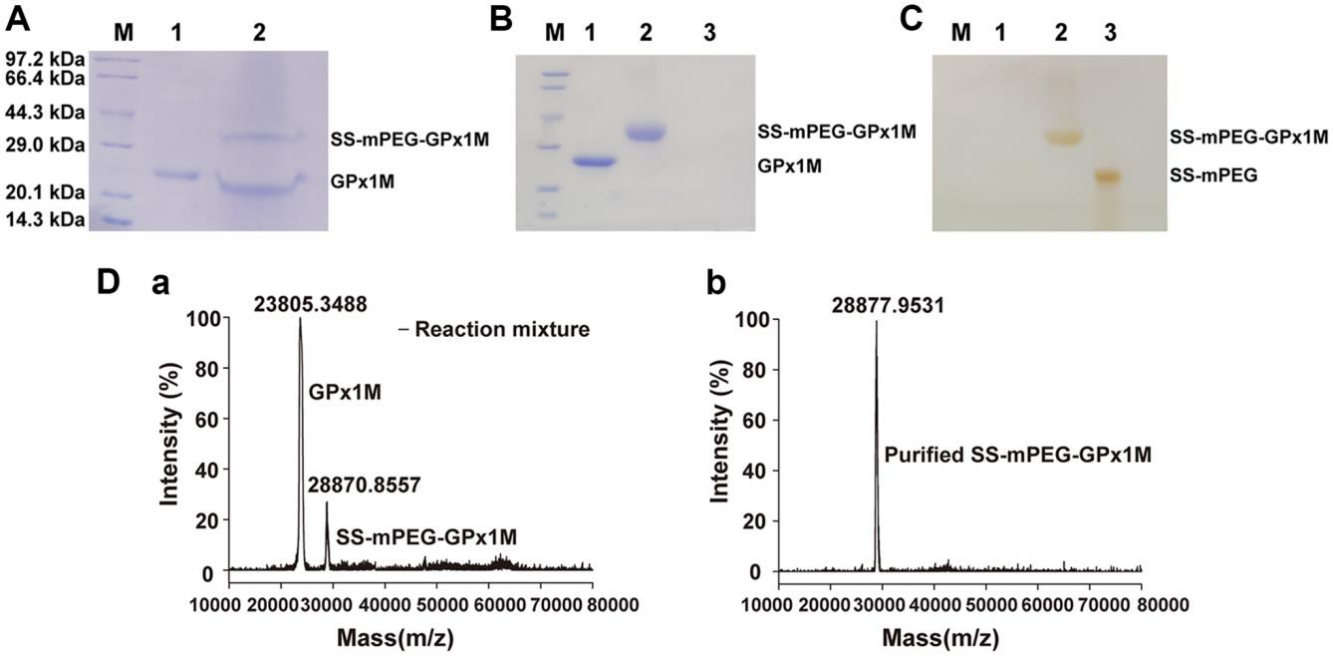
**Characterization of SS-mPEG-GPx1M.** (**A**) SS-mPEG-GPx1M form at mass ratio 1:50 stained with Coomassie Blue R-250. M, molecular weight standards. Lane 1, GPx1M. Lane 2, the reaction mixture with a molar ratio of 1:50 between GPx1M and SS-mPEG, including unmodified GPx1M and SS-mPEG-GPx1M. SDS-PAGE analysis of purified SS-mPEG-GPx1M with Coomassie Blue R-250 (**B**) and Iodine stain (**C**). Abbreviation: M: molecular weight standards. Lane 1, GPx1M. Lane 2, purified SS-mPEG-GPx1M. Lane 3, SS-mPEG. (**D**) MALDI-TOF-MS of reaction mixture and purified SS-mPEG-GPx1M. (**a**) The MS image of reaction mixture. The reaction mixture mainly included unmodified GPx1M (23805.3488 m/z) and PEGylated product (SS-mPEG-GPx1M, 28870.8557 m/z). (**b**) The MS image of purified SS-mPEG-GPx1M.

The predicted molecular weights from the SDS-PAGE results indicated that the GPx1M conjugate contains a single PEG molecule (Figure 2A). Analysis of Figure 2A by ImageJ showed that the ratio of SS-mPEG-GPx1M to unmodified GPx1M was 1:1.83, and the modification rate of SS-mPEG-GPx1M was 35.33%. The purification result of SS-mPEG-GPx1M was observed by SDS-PAGE analysis in Figure 2B–2C.

To analyze the molecular mass distribution of GPx1M with SS-mPEG, MALDI-TOF-MS was applied to confirm the SDS-PAGE results (Figure 2D). In the MALDI-TOF-MS result (a), 23805.3488 m/z was GPx1M, and 28870.8557 m/z was SS-mPEG-GPx1M. The result showed that GPx1M was successfully PEGylated with SS-mPEG. In the MALDI-TOF-MS result (b), 28877.9531 m/z was the purified SS-mPEG-GPx1M, and there was only one peak in the MS, indicating that the purified product has been obtained with high purity.

**Figure 3:**
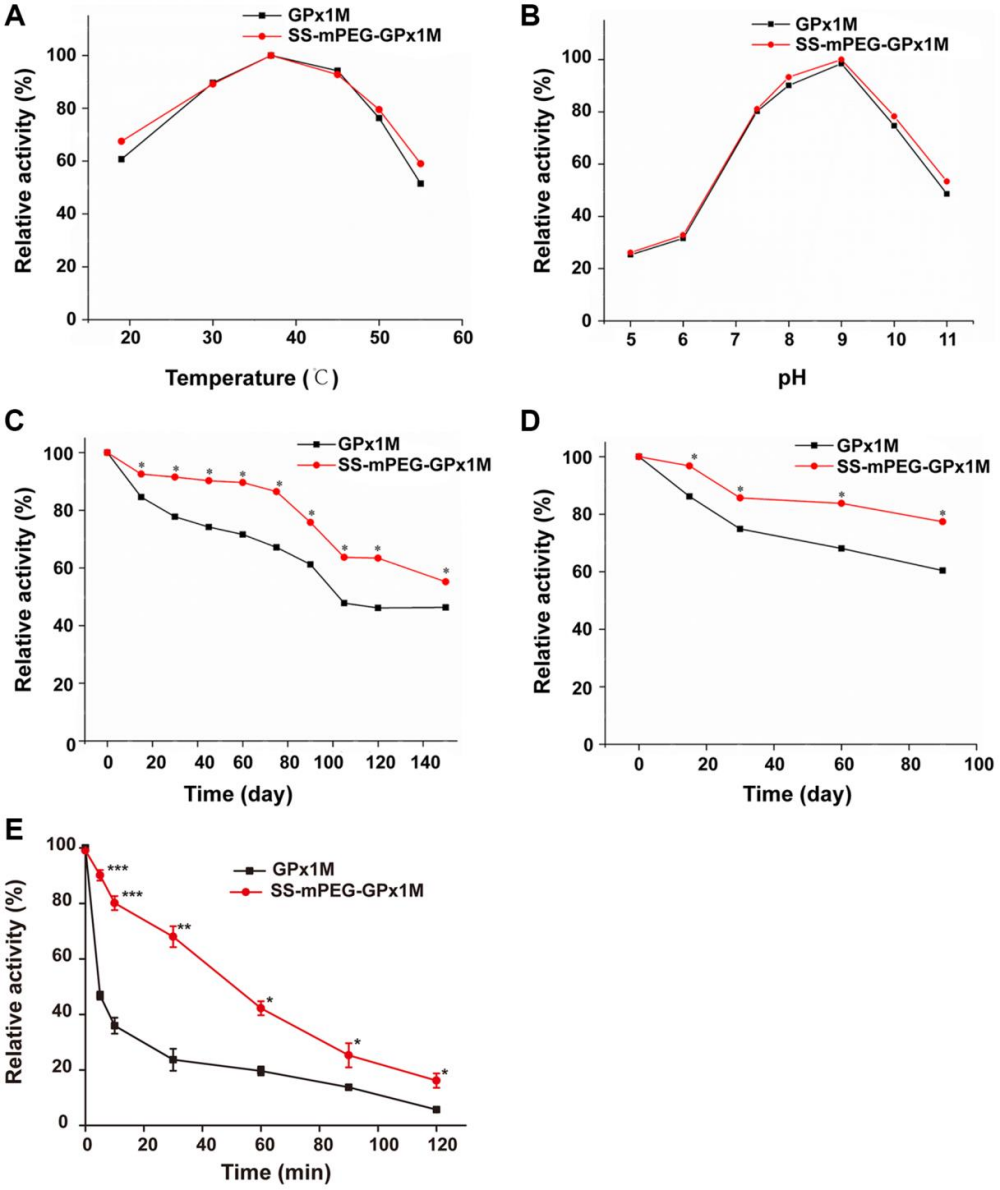
**Determination of relative enzyme activity of SS-mPEG-GPx1M.** Temperature (**A**) and pH (**B**) profiles of GPx1M and SS-mPEG-GPx1M. (**C**) GPx1M and SS-mPEG-GPx1M were incubated in 50 mM PBS at 4°C for a period ranging from 1 to 150 days. (**D**) GPx1M and SS-mPEG-GPx1M were incubated in 50 mM PBS at −20°C for a period ranging from 1 to 90 days. (**E**) The resistance of SS-mPEG-GPx1M to protease hydrolysis. All values were converted to a relative value, and the highest enzyme activity was set as 100%. ^*^p < 0.05, ^**^p < 0.01, and ^***^p < 0.001 vs. GPx1M.

The temperature profiles of GPx1M and SS-mPEG-GPx1M were shown in Figure 3A. The results revealed that both GPx1M and SS-mPEG-GPx1M presented a similar optimum temperature at 37°C. SS-mPEG-GPx1M exhibited a slight improvement in its thermal stability between 50–55°C. GPx1M exhibited 51.44% enzyme activity at 55°C, whereas SS-mPEG-GPx1M retained 59.04% activity under the same conditions. The pH profiles for GPx1M and SS-mPEG-GPx1M presented an optimal activity peak at pH 9.0 (Figure 3B). The enzyme activity of SS-mPEG-GPx1M was higher than free GPx1M over the studied pH values. The results indicated that the PEGylated strategy offered a method to protect against enzyme inactivation and improve the pH and thermal stability of GPx1M.

The effects of storage temperatures on SS-mPEG-GPx1M and GPx1M were determined. SS-mPEG-GPx1M and GPx1M had similar enzyme activity behaviors under the different temperature conditions, with a gradual decrease in activity during the storage period. However, there were statistically significant changes in the relative activity over the storage periods for SS-mPEG-GPx1M and GPx1M at different temperatures. The results showed that the relative activity of SS-mPEG-GPx1M was superior to that of GPx1M at 4°C and −20°C. After exposure for 150 days at 4°C, GPx1M and SS-mPEG-GPx1M retained 46.32% and 55.21% activity, respectively (Figure 3C). SS-mPEG-GPx1M showed 77.39% enzyme activity after 90 days at −20°C, whereas GPx1M retained 60.42% (Figure 3D). The above results demonstrated that PEGylation is an effective method to improve the long-term stability of GPx1M.

The results of the resistance of the modified product to protease could be observed in Figure 3E. The results showed that the sensitivity of SS-mPEG-GPx1M to protease was significantly lower than that of GPx1M, indicating that PEGylation could increase the resistance of GPx1M to protease hydrolysis.

### PEGylation site

**Figure 4:**
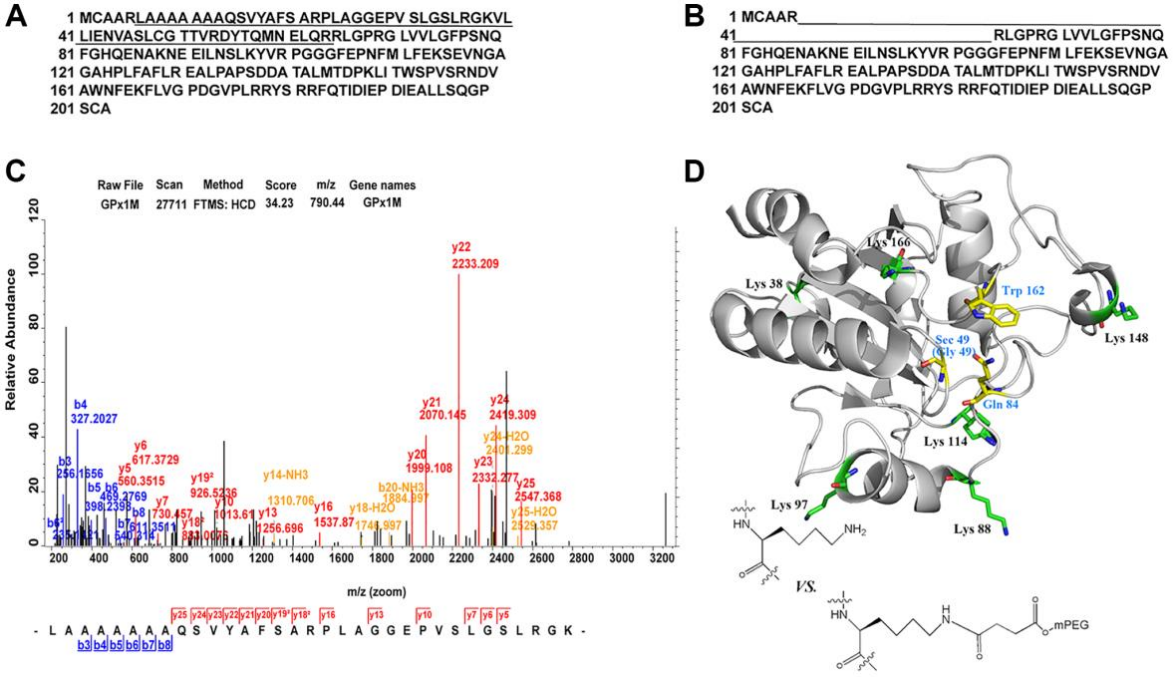
**Identification of PEGylation site and spatial structure of GPx1M.** (**A**) The peptide sequences derived from GPx1M digestions with trypsin. (**B**) The peptide sequences derived from SS-mPEG-GPx1M digestions with trypsin. (**C**) The ESI-MS analysis of peptides from digested GPx1M. (**D**) The structure of GPx1M with side chains shown as sticks.

All digestive products of SS-mPEG-GPx1M treated with trypsin were compared with digestions of GPx1M, and the peptide sequences LAAAAAAAQSVYAFSARPLAGGEPVSLGSLRGK and VLLIENVASLCGTTVRDYTQMNELQR with missed cleavage site were only detected from the digestions of GPx1M (Figure 4A–4B). The application guide of MaxQuant software pointed out that the number of missing cleavage sites was allowed to be 1 or 2, and the phenomenon of missing cleavage caused by trypsin was common in protein identification. The researchers used a data set composed of multiple proteins to prove that when lysine and arginine were adjacent to each other or were close, missing cleavage would occur. Meanwhile, when lysine and arginine were adjacent to aspartic acid, glutamic acid, or proline, the missing cleavage would also occur [22, 23]. Moreover, in this study, the length of the two peptides did not exceed 33 amino acids, and both were within the range of mass spectrometry identification, so this result was normal and credible. The more information of the peptide sequences of LAAAAAAAQSVYAFSARPLAGGEPVSLGSLRGK was provided by ESI-MS (Figure 4C). The spatial structure of GPx1M with possible binding sites for PEGylated lysine residues were highlighted in green (Lys 38, Lys 88, Lys 97, Lys 114, Lys 148, and Lys 166) and the active site center was highlighted in yellow (Sec 49, Gln 84, and Trp 162) (Figure 4D). The structure was based on the impact of PEGylation on conformational stability. The models were made using hGPx1 (PDB ID code 2F8A) and visualized with PyMOL and Chemdraw. Through analysis, we finally determined that Lys 38 was the PEGylation site.

### Pharmacokinetic analysis

**Figure 5:**
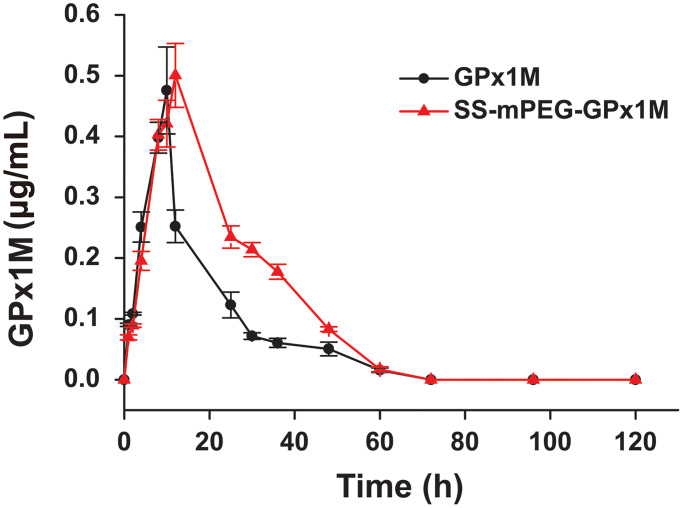
**Blood concentration of GPx1M and SS-mPEG-GPx1M.** All data were showed as mean ± SD (*n* = 4).

The concentration of GPx1M in the GPx1M group decreased rapidly after reaching the highest blood concentration (*C*_max_), but the concentration of SS-mPEG-GPx1M in the SS-mPEG-GPx1M group decreased slowly (Figure 5). By analyzing pharmacokinetic parameters (Table 1), we found that the blood circulation half-life (*t*_1/2_) of the SS-mPEG-GPx1M group was 1.6-fold that of the GPx1M group, and the renal clearance rate (*Cl/F*) was 64%. The area under the curve (*AUC*_0–120 h_) of the SS-mPEG-GPx1M group was obviously increased compared to the GPx1M group. Data showed that the modification significantly increased GPx1M retention time while reducing renal clearance.

**Table 1:** Pharmacokinetic parameters of SS-mPEG-GPx1M in SD rats.

**Sample**	***t*_1/2_^a^ (h)**	***C*_max_^b^ (mg/L)**	***AUC*_0–120 h_^c^ (mg/L/h)**	***Cl/F*^d^ (mL/h/kg)**
GPx1M	14.73 ± 1.72	0.48 ± 0.06	7.95 ± 0.72	12.26 ± 1.11
SS-mPEG-GPx1M	24.04 ± 0.39^**^	0.51 ± 0.04	12.58 ± 0.50^**^	7.84 ± 0.34^**^

^a^The blood circulation half-life. ^b^The maximum blood concentration. ^c^The area under the curve. ^d^The clearance rate. ^**^p < 0.01 vs. GPx1M.

### *In vitro* antioxidant effect analysis

**Figure 6:**
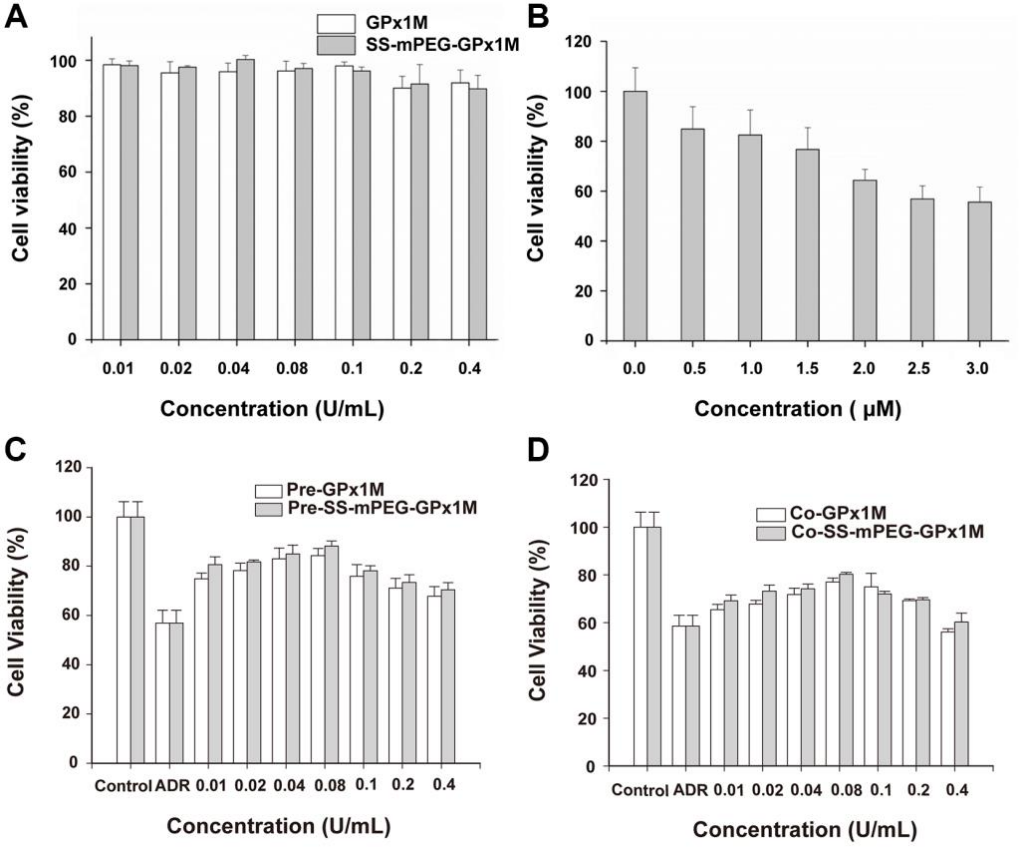
**Determination of cell viability *in vitro*.** Cytotoxic effects of cotreatment with GPx1M, SS-mPEG-GPx1M (**A**), and ADR (**B**) on H9c2 cells. (**C**) Cell viabilities of H9c2 cells that were preincubated with different concentrations of GPx1M and SS-mPEG-GPx1M (0.01–0.4 U/mL) for 1 h, and then treated with ADR (2.5 μM) for 24 h. (**D**) Cell viabilities of H9c2 cells that were treated with ADR (2.5 μM) for 12 h, and then co-incubated with different concentrations of GPx1M and SS-mPEG-GPx1M (0.01–0.4 U/mL) for another 12 h.

We evaluated the effects of different concentrations of GPx1M and SS-mPEG-GPx1M (0.01–0.4 U/mL) on H9c2 cell cytotoxicity to obtain an optimal concentration of GPx1M and SS-mPEG-GPx1M which is both innocuous to cells and effective for alleviating ADR-induced cytotoxicity. The different concentrations of GPx1M and SS-mPEG-GPx1M (0.01–0.1 U/mL) were not cytotoxic toward H9c2 cells (Figure 6A). H9c2 cell viability exhibited a slightly decrease at the 0.2–0.4 U/mL concentrations. However, ADR-induced cytotoxicity was dose-dependent. The viability of H9c2 cells treated with 2.5 μM ADR was 56.88 ± 5.27% (Figure 6B). This drug concentration was set as the model concentration for further investigation.

The results of pre-/cotreatment of GPx1M and SS-mPEG-GPx1M on ADR-induced injury in H9c2 cells indicated that both pre-/coincubation of GPx1M and SS-mPEG-GPx1M notably decreased cytotoxicity and increased cell viability (Figure 6C–6D). Moreover, SS-mPEG-GPx1M exhibited slightly enhanced protective effects than GPx1M on H9c2 cells. The result revealed that pre-/cotreatment with 0.08 U/mL of GPx1M (84.06 ± 3.01%/76.19 ± 1.17%) and SS-mPEG-GPx1M (86.65 ± 1.87%/79.82 ± 0.60%) could alleviate ADR-induced damage and improve H9c2 cell viability. Based on the above results, we selected 0.08 U/mL GPx1M and SS-mPEG-GPx1M for further study.

**Figure 7:**
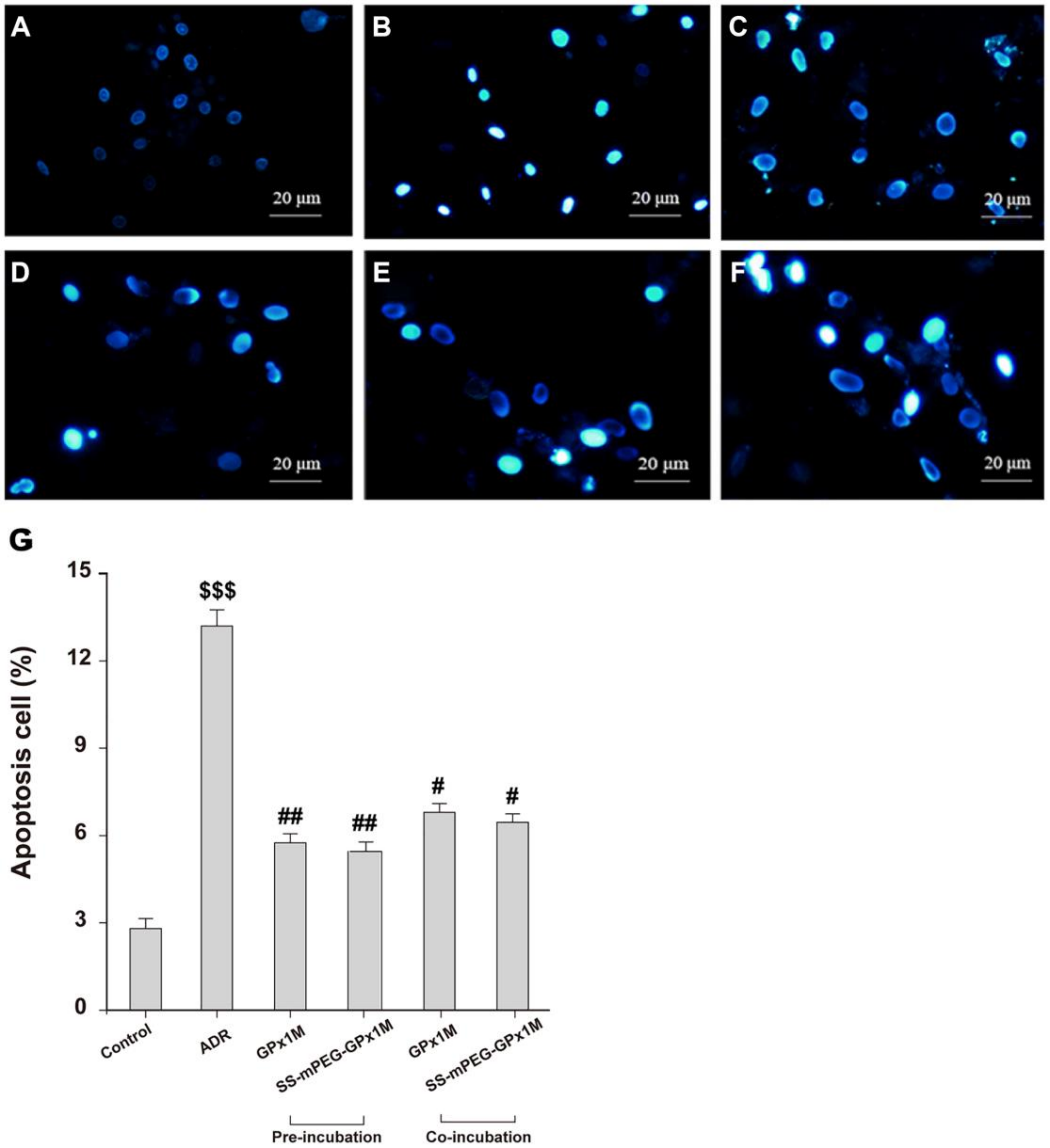
**Effects of GPx1M and SS-mPEG-GPx1M on ADR-induced H9c2 apoptosis evaluated by Hoechst 33258 staining using a fluorescence microscope (200×).** The experiments were repeated in triplicate and representative images were shown. Cells were treated with: (**A**) Control (DMEM). (**B**) 2.5 μM ADR. (**C**) and (**D**) cells preincubated with GPx1M/SS-mPEG-GPx1M (0.08 U/mL) for 1 h, respectively, and then co-incubated with 2.5 μM ADR for 24 h. (**E**) and (**F**) cells incubated with 2.5 μM ADR for 12 h, then in co-incubated with GPx1M/SS-mPEG-GPx1M (0.08 U/mL), respectively, for another 12 h. (**G**) Apoptotic H9c2 cells incubated with different methods. All data were exhibited as mean ± SD. ^$$$^*p* < 0.001 vs. the control group, ^#^*p* < 0.05 and ^##^*p* < 0.01 vs. the ADR group.

The reduction in cell viability might be attributed to ADR-induced apoptosis in H9c2 cells. Figure 7 showed the morphological changes of apoptotic cells stained with Hoechst 33258, exhibiting nuclei condensation and fragment staining. Hoechst 33258 staining revealed that exposure to ADR reduced the cell viability, and apoptotic cells appeared to be white in color. Pre-/coincubation with GPx1M and SS-mPEG-GPx1M led to fewer white-colored cells and an obvious decrease in the number of apoptotic cells compared to the ADR group.

**Figure 8:**
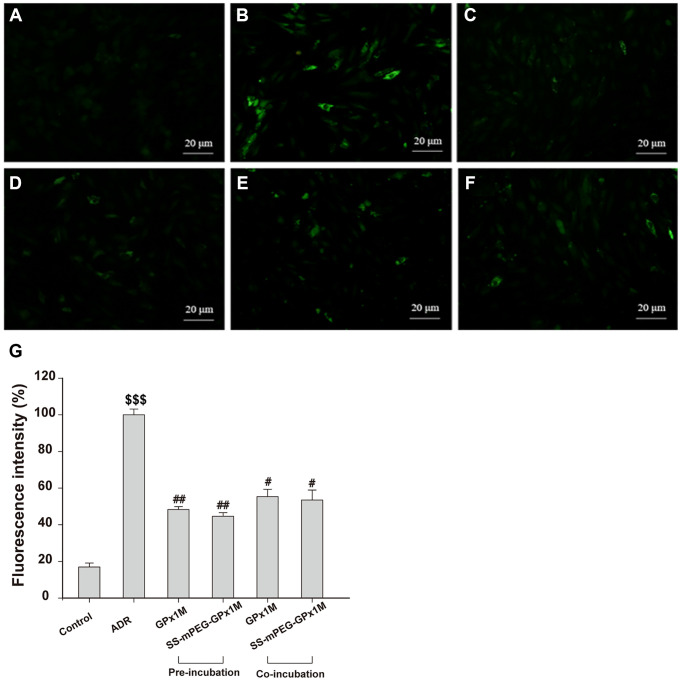
**Effects of GPx1M and SS-mPEG-GPx1M on ADR-induced generation of ROS in H9c2 cells.** Cells were treated with: (**A**) Control (untreated). (**B**) 2.5 μM ADR. (**C**) and (**D**) cells preincubated with GPx1M/SS-mPEG-GPx1M (0.08 U/mL) for 1 h, respectively, and then in co-incubated with 2.5 μM ADR for 24 h. (**E**) and (**F**) cells incubated with 2.5 μM ADR for 12 h, and then co-incubated with GPx1M/SS-mPEG-GPx1M (0.08 U/mL), respectively, for another 12 h. The cells were stained with DCFH-DA and analyzed with a fluorescence microscope (**G**). The experiments were repeated in triple and results were shown as the mean ± SD. ^$$$^*p* < 0.001 vs. the control group, ^#^*p* < 0.05 and ^##^*p* < 0.01 vs. the ADR group.

ADR-mediated redox cycling caused an increase in the generation of ROS and cell damage [24]. Figure 8 revealed the effects of ROS generation in ADR-, GPx1M-, and SS-mPEG-GPx1M-treated cells. Incubation with ADR showed an obvious increase in ROS generation as indicated by the 5.90-fold increase in fluorescence intensity compared to the normal group. H9c2 cells pre-/co-administrated GPx1M (48.34 ± 1.62% and 55.43 ± 3.91%) and SS-mPEG-GPx1M (44.64 ± 1.97% and 53.54 ± 5.39%) followed by ADR illustrated a significant decrease in ROS production compared to H9c2 cells incubated with ADR alone (100 ± 3.12%). The abilities of GPx1M and SS-mPEG-GPx1M to decrease ROS accumulation in cardiomyoblasts were attributed to their ability to scavenge free radicals.

**Figure 9:**
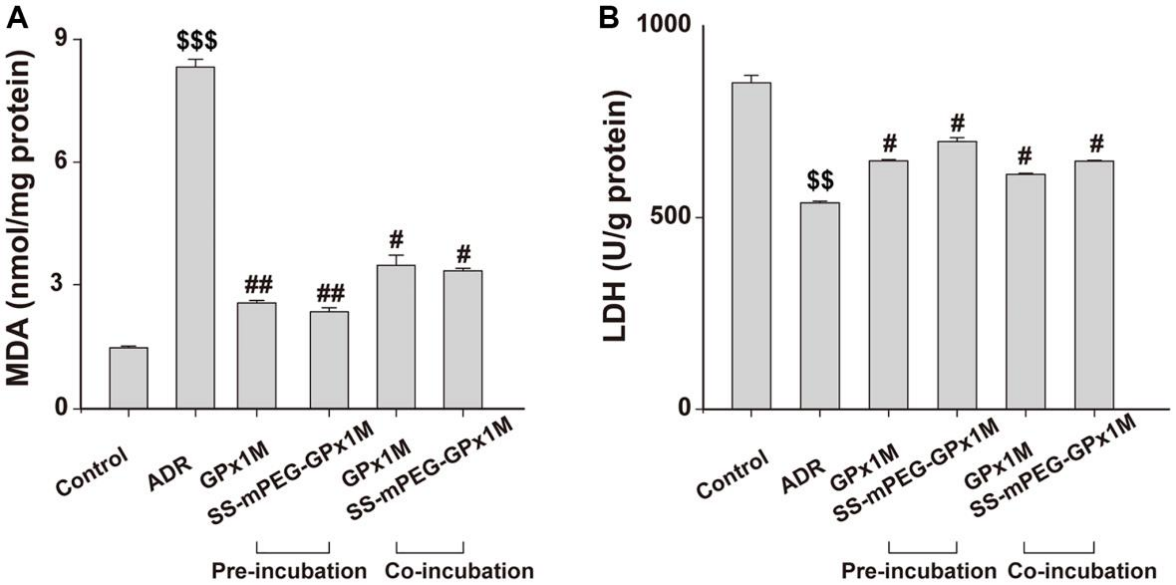
Effects of GPx1M and SS-mPEG-GPx1M on MDA (**A**) and LDH release (**B**) from H9c2 cells incubated with ADR. Cells were either preincubated with GPx1M/SS-mPEG-GPx1M (0.08 U/mL) for 1 h and then co-incubated with 2.5 μM ADR for 24 h or incubated with 2.5 μM ADR for 12 h and then co-incubated with GPx1M/SS-mPEG-GPx1M (0.08 U/mL) for another 12 h. ^$$^*p* < 0.01 and ^$$$^*p* < 0.001 vs. the control group, ^#^*p* < 0.05 and ^##^*p* < 0.01 vs. the ADR group.

The lipid peroxidation contents were assayed by detecting MDA, which is the final product in lipid peroxidation. Compared with the control group, a significant increase in MDA was observed in the ADR group. Additionally, the MDA contents were decreased in the cells pre-/co-incubated with GPx1M (2.55 ± 0.06 nM/mg and 3.45 ± 0.25 nM/mg) and SS-mPEG-GPx1M (2.34 ± 0.09 nM/mg and 3.32 ± 0.06 nM/mg) compared to the ADR group (Figure 9A). These results indicated that GPx1M and SS-mPEG-GPx1M could alleviate the oxidative stress mediated by ADR in H9c2 myoblasts.

The intracellular cell LDH contents in the ADR group (538.41 ± 4.59 U/g) were obviously decreased compared with the control group (851.73 ± 18.60 U/g). The LDH contents after preincubation with GPx1M and SS-mPEG-GPx1M were 647.76 ± 3.92 U/g and 698.68 ± 9.50 U/g, respectively. Furthermore, H9c2 cells co-incubated with GPx1M and SS-mPEG-GPx1M resulted in an obvious increase in LDH (612.42 ± 2.82 U/g and 647.05 ± 2.11 U/g, respectively) compared to the ADR group (Figure 9B). The data showed that GPx1M and SS-mPEG-GPx1M could prevent cell membranes from undergoing oxidative stress damage.

### *In vivo* antioxidant effect analysis

**Figure 10:**
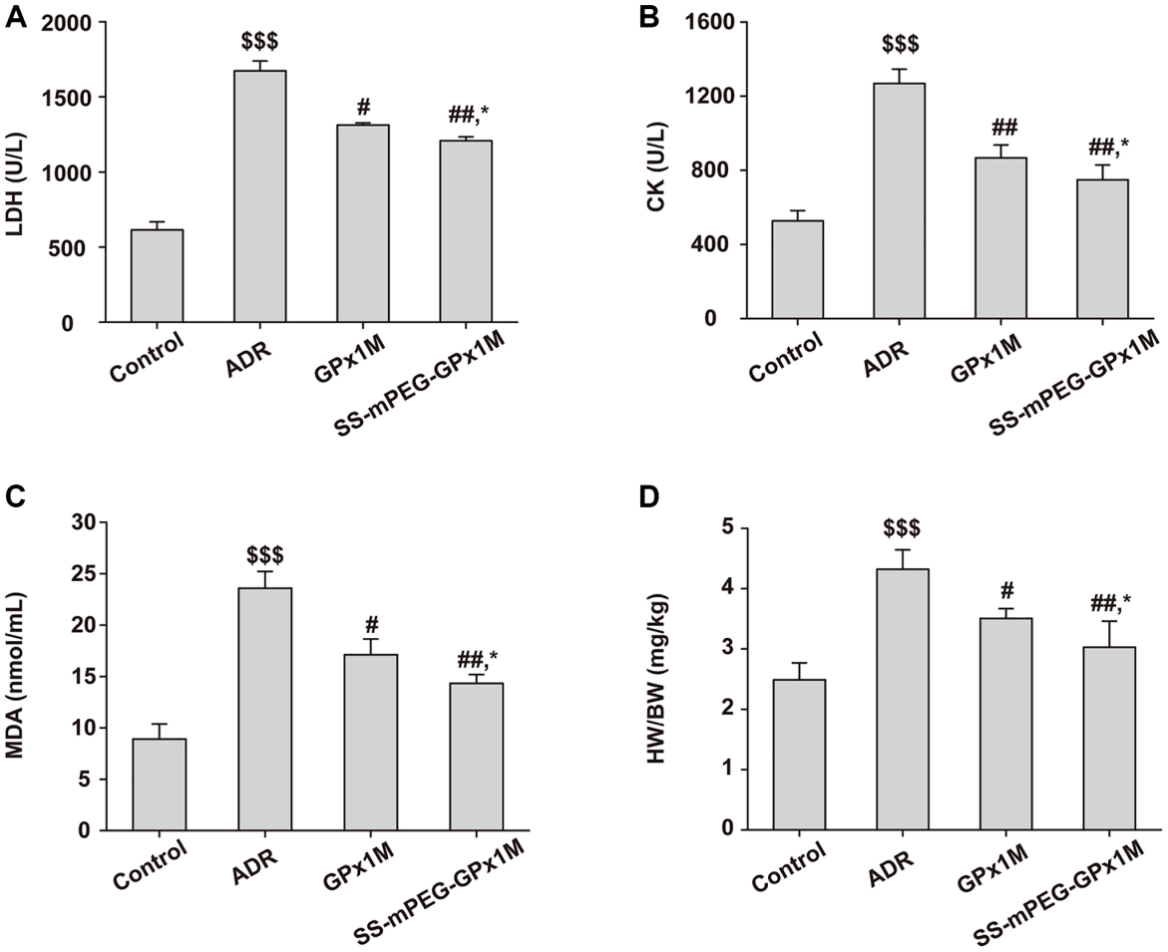
**Antioxidant effect analysis of SS-mPEG-GPx1M *in vivo*.** The levels of LDH (**A**) and CK (**B**) in serum were determined. The content of MDA (**C**) in serum and the heart weight/body weight ratios (HW/BW) (**D**) of all groups were analyzed. All data were exhibited as mean ± SD (*n* = 5). ^$$$^*p* < 0.001 vs. the control group, ^#^*p* < 0.05 and ^##^*p* < 0.01 vs. the ADR group, and ^*^p < 0.05 vs. the GPx1M group.

The cardiac enzymes analysis of LDH (Figure 10A) and CK (Figure 10B) illustrated similar results. The content of cardiac enzymes in the control group were normal. On the other hand, the levels of these enzymes in the ADR group increased significantly. Compared with the control group, the index values of the GPx1M group and the SS-mPEG-GPx1M group were increased. The contents of LDH and CK in the GPx1M and SS-mPEG-GPx1M group decreased significantly compared with that of the ADR group. The values of these substances in the SS-mPEG-GPx1M group were lower than those in the GPx1M group.

The results of MDA were shown in Figure 10C. Compared with the control group, the content of MDA in the ADR group was significantly incremental. The levels of MDA in the GPx1M and SS-mPEG-GPx1M group were the opposite of the ADR group. The content of MDA in the GPx1M group was higher than that in the SS-mPEG-GPx1M group.

The HW/BW of all groups suggested that the ADR group caused an obvious improvement of HW/BW compared with the control group (Figure 10D). Compared to the ADR group, rats of the GPx1M group and the SS-mPEG-GPx1M group exhibited significant reduced of HW/BW. Moreover, the HW/BW of the GPx1M group was higher than that of the SS-mPEG-GPx1M group.

**Figure 11:**
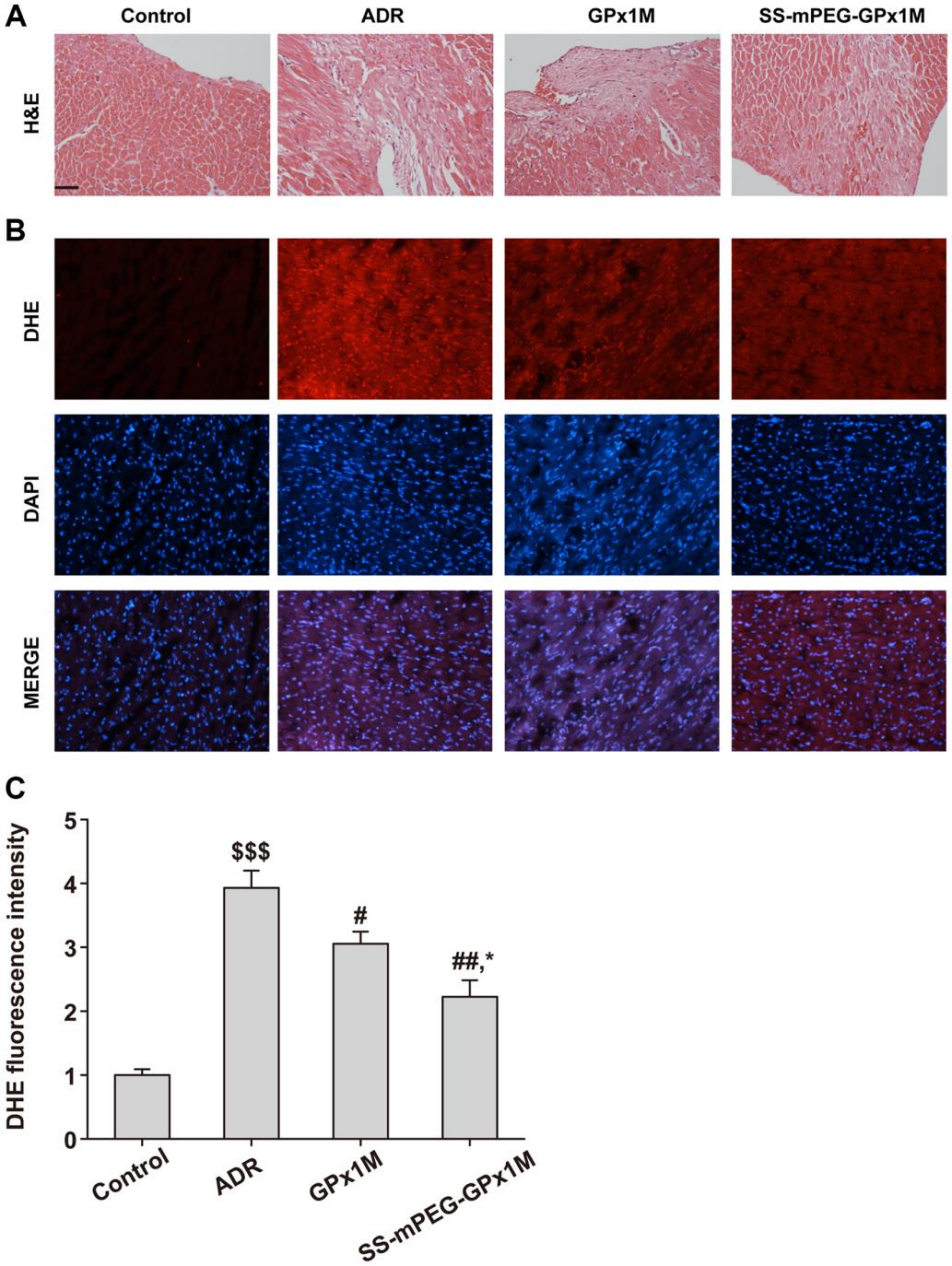
**Histopathological changes and ROS measurement in all groups.** (**A**) Analysis of histological evaluation in rat heart tissue using H&E. (**B**) Determination the accumulation of ROS in rat heart by DHE staining. (**C**) DHE fluorescence intensity of each group. Scale bars, 100 μm. All data were exhibited as mean ± SD. ^$$$^*p* < 0.001 vs. the control group, ^#^*p* < 0.05 and ^##^*p* < 0.01 vs. the ADR group, and ^*^p < 0.05 vs. the GPx1M group.

H&E results showed that the ADR group had obvious myocardial damage, nuclear membrane disappearance, and cell deformation, while the GPx1M group and the SS-mPEG-GPx1M group had different degrees of relief compared with the ADR group, and the alleviating effect of the SS-mPEG-GPx1M group was more obvious than the GPx1M group (Figure 11A).

DHE results showed that the ADR group had obvious DHE fluorescence intensity, indicating that there was a large amount of ROS in the myocardial tissue of the ADR group compared with the control group. Compared with the ADR group, the ROS content in the heart of the GPx1M group and the SS-mPEG-GPx1M group was significantly reduced, and the accumulation of ROS in the SS-mPEG-GPx1M group was less than that of the GPx1M group (Figure 11B–11C).

The above results indicated that GPx1M and SS-mPEG-GPx1M had antioxidant effects *in vivo*, in which the antioxidant function of SS-mPEG-GPx1M was stronger than that of GPx1M. Moreover, the half-life of GPx1M for *in vivo* antioxidant therapy after PEGylation was improved. This was mainly due to the slow release, the prolonged serum half-life, and the enhanced ability to resist protease hydrolysis of GPx1M after PEGylation which increased the cumulative amount of the drug *in vivo*, and ultimately enhanced the half-life of GPx1M for *in vivo* antioxidant therapy.

## DISCUSSION

PEGylation is an efficient method to improve enzyme stability [20]. PEGylation can increase the particle size, enhance stability, and prevent effects due to temperature, pH, and proteolytic digestion [21]. GPx1M has six lysine residues available for potential PEGylation. Based on the structure-function relationships between glutathione and GPx1M, Lys 88 and Lys 166 are near the GPx1M catalytic active center (Sec 49, Gln 84, and Trp 162), which might reduce its enzymatic activity. However, the results indicated that PEGylation of GPx1M did not decrease its enzymatic activity. There might be two reasons for this phenomenon. One reason may be due to the attachment of the PEG side chain to lysine residues remote from the catalytic center. Thus, the PEG chains will not prevent the access of binding partners or substrates to the protein’s catalytic pocket [25]. Another reason may be partially attributed to the fact that glutathione is a small substrate molecule [26], which allows GPx1M to maintain its enzymatic activity. Thus, the SS-mPEG-GPx1M conjugate suggested a similar maximum enzyme activity at 37°C and pH 9.0 to GPx1M in the *in vitro* enzymatic assays. Moreover, SS-mPEG-GPx1M had a slightly increased enzyme activity with respect to thermal stability and pH stability. The increase in SS-mPEG-GPx1M long-term stability (4°C and −20°C) might be attributed to the reduction of positive electrostatic charges, which decreased the charge exclusion within the protein [27]. Additionally, GPx1M was surrounded by PEG groups, which generated a condensed natural conformation. This alteration meant the tensile strength could not be easily stretched, leading to a decrease in the thermal vibration of enzyme molecules. In addition, PEGylation decreased the hydrophobicity of the protein surface, which made it produce more salt bridges and hydrogen bonds in the water medium and thus improved the thermal and long-term stability of GPx1M [28].

ADR could acquire one electron nonenzymatically from cytochrome P450 or through enzymatically catalyzed conditions and then be converted into its semiquinone form. This semiquinone form could be oxidized to its quinone structure with a concomitant generation of superoxide free radicals [29]. ROS could directly damage mitochondria in cardiomyocytes which inducing cardiotoxicity [30]. Radicals are produced continuously in the human body as a result of serious diseases [31]. Due to the extensive use of ADR in clinical studies, researchers worldwide have attempted to decrease its severe side effects. However, the present methods (analogue drugs or combined treatment) have been proven to be mostly ineffective [32].

GPx is one of the important antioxidant enzymes (superoxide dismutase, catalase, and GPx). Natural hGPx1 exhibits significant radical scavenging activity, which might be a potential candidate for alleviating ADR-induced cardiotoxicity. GPx overexpression can decrease lipid peroxidation contents in ADR-treated human breast carcinoma cells [33], revealing that the suppression of GPx is closely related to apoptosis [34]. Thus, H9c2 cells were selected as model cells to evaluate ADR-induced cardiotoxicity [35]. We observed that ADR showed a prominent and concentration-dependent toxicity in H9c2 cells. However, different concentrations of GPx1M and SS-mPEG-GPx1M (0.01–0.1 U/mL) did not present any cytotoxicity. Pre-/coincubation of GPx1M and SS-mPEG-GPx1M notably decreased the cytotoxicity and increased cell viability. Moreover, SS-mPEG-GPx1M exhibited similar protective effects to GPx1M on H9c2 cells.

Apoptosis results revealed that H9c2 cells administered ADR presented a large number of white-colored cells compared with the control group. Cells pre-/cotreated with GPx1M and SS-mPEG-GPx1M showed a notable decrease in white-colored cells. GPx1M could alleviate ADR-induced cytotoxicity [33]. The alleviation effects of GPx1M were also reported in other investigations, which were due to its anti-apoptotic, anti-lipid peroxidation and antioxidant effects. ADR-mediated redox cycling caused an increase in the generation of ROS and cell damage [24]. Pre-/cotreatment with GPx1M and SS-mPEG-GPx1M could decrease ROS production as evident by the lower fluorescent intensity. The abilities of GPx1M and SS-mPEG-GPx1M to decrease ROS accumulation in cardiomyoblasts were attributed to their ability to scavenge free radicals.

The reduction in MDA showed that GPx1M and SS-mPEG-GPx1M could alleviate the oxidative stress mediated by ADR in H9c2 myoblasts. They function by preventing lipid peroxidation and ROS generation. GPx1M and SS-mPEG-GPx1M could catalyze the attenuation of hydrogen peroxide and other lipid peroxides, while SOD could catalyze superoxide free radicals to hydrogen peroxide [36]. Based on these reasons, the MDA contents were reduced by GPx1M and SS-mPEG-GPx1M.

**Figure 12:**
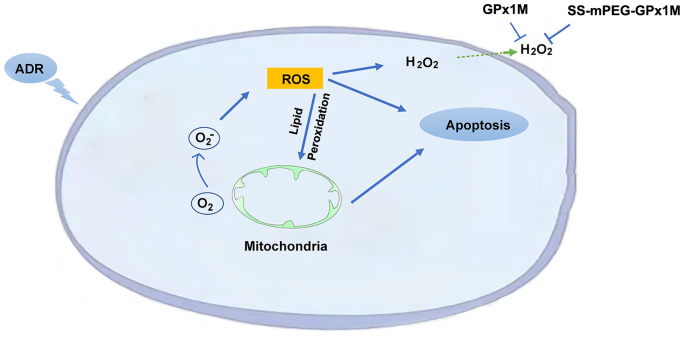
Schematic illustration of the mechanism of SS-mPEG-GPx1M against ADR-induced oxidative stress injury and cardiomyocyte apoptosis.

The presence of lipid peroxidation can lead to cardiomyoblast membrane damage, allowing intracellular components to leak into the medium. LDH is an intracellular enzyme detected following damage to cell membranes and is an important indicator of cell membrane integrity. GPx1M and SS-mPEG-GPx1M could prevent cell membranes from undergoing oxidative stress damage. GPx1M and SS-mPEG-GPx1M could reduce the oxidative stress-mediated cytotoxicity, which may be due to their ability to scavenge a variety of fatty acid hydroperoxides and decrease H_2_O_2_ production, which in turn decreases apoptosis as well as ROS production (Figure 12).

Studies on antioxidant activity *in vivo* showed that the levels of cardiac enzymes (LDH and CK), MDA in serum and HW/BW of the GPx1M group and the SS-mPEG-GPx1M group were lower than the ADR group. Data indicated the antioxidant effect of SS-mPEG-GPx1M was stronger than that of GPx1M *in vivo*. This may be that PEGylation could increase the molecular weight of GPx1M, so that is not easily removed by the kidney. On the other hand, PEG could cover the surface of GPx1M, and reduce the exposure of antigenic determinants. As a result, the modified GPx1M could remain in the body for a longer time which caused a prolonged biological half-life, and had more opportunities to eliminate excess reactive oxygen species.

In conclusion, the stability, the half-life and the antioxidant effect *in vivo* of the GPx1M was significantly improved after PEGylation while the remaining enzyme activity after modification was basically the same as GPx1M. After modification, SS-mPEG-GPx1M still had an equivalent antioxidant capacity with GPx1M *in vitro*, which did not affect the function of GPx1M. In this research, we successfully synthesized methoxypolyethylene glycol-succinimidyl succinate and prepared a novel SS-mPEG-GPx1M polymer through covalent attachment. SS-mPEG-GPx1M showed improved temperature stability at 50–55°C and pH stability at 10–11. The enzymatic activity of SS-mPEG-GPx1M at different storage temperatures, namely, 4°C and −20°C, was superior to that of GPx1M. SS-mPEG-GPx1M exhibited similar antioxidant effects to GPx1M by decreasing MDA production, inhibiting LDH release and alleviating ADR-induced H9c2 cells damage. Meanwhile, SS-mPEG-GPx1M had better antioxidant activity and longer blood circulation half-life than GPx1M *in vivo*. Thus, PEGylation could improve the stability of GPx1M and prompt the application of GPx1M as an important antioxidant and potential drug.

## MATERIALS AND METHODS

### Materials

Methoxy polyethylene glycols were purchased from Sigma Aldrich (USA). MTT (3-(4, 5-dimethylthiazol-2-yl)-2, 5-diphenyl tetrazolium bromide), succinic anhydride (SA), N, N dimethyl formamide (DMF), 1-ethyl-3-[3-(dimethyl amino) propyl] carbodiimide (EDC), and N-hydroxysuccinimide (NHS) were supplied by Energy Chemical Co., Ltd. (Shanghai, China). Diethyl ether and dichloromethane were purchased from Sinopharm Chemical Reagent Co., Ltd. (Beijing, China). Fetal bovine serum (FBS), Dulbecco’s modified Eagle medium (DMEM), trypsin, and phosphate buffer saline (PBS) were all supplied by Thermo Fisher Scientific Co., Ltd. (USA). LDH, creatine kinase (CK), and MDA assay kits were provided by Nanjing Jiancheng Bioengineering Institute (Nanjing, China). Hoechst 33258 staining kit and reactive oxygen species (ROS) assay kits were supplied by Beyotime biotechnology (Jiangsu, China).

### Animals

All animals were obtained from the Laboratory Animal Center of Jilin University (Changchun, China) and housed on 12-h light, 12-h dark cycle with commercial food and water. Animal experiments were carried out following the Committee for the Care and Use of Laboratory Animals of Jilin University (Changchun, China) and the Guide for the National Institutes of Health guide for the care (NIH Publication No. 8023, revised 1978).

### Synthesis and characterization of SS-mPEG

**Scheme 1:**
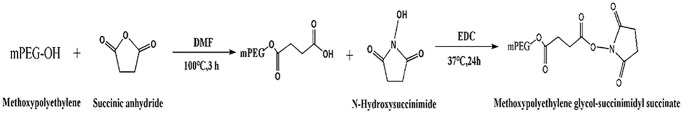
Synthetic route of methoxypolyethylene glycol-succinimidyl succinate.

SA, NHS, EDC, and mPEG were used to synthesize SS-mPEG (Scheme 1). Certain amounts of SA (0.363 g), mPEG (6.002 g), and DMF (12 mL) were placed into a reaction bottle. The bottle was immersed in an oil bath and stirred at 100°C for 3 h. NHS (1.400 g) and EDC (2.478 g) were added to the mixture solution at room temperature and maintained at 37°C for 24 h. The obtained mixture solution was precipitated with diethyl ether (120 mL) at 4°C. The raw product was dissolved in dichloromethane and precipitated with diethyl ether 4 times. SS-mPEG was dried in vacuum and the yield was 5.375 g.

^1^H-NMR spectra of the samples were obtained with a Bruker AVIII NMR spectrometer at 25°C using DMSO-*d*_6_ as the solvent and tetramethylsilane as the reference [37].

### Modification and purification of SS-mPEG-GPx1M

GPx1M (Seleno-hGPx1-C78/115/156S) was obtained from our previous study [4]. Conjugation of GPx1M with SS-mPEG was performed per Sakakibara et al. [38]. SS-mPEG and GPx1M were mixed with 1 mL of 50 mM PBS (pH 8.5) in a tube. The mass ratio of GPx1M to SS-mPEG was 1:50. The protein solution was stirred at 80 rpm/min for 30 min at 4°C. The reaction mixture was placed into a dialysis bag and dialyzed with 1 L of 50 mM PBS (pH 7.4) three times over 4 h at 4°C. The DEAE-sephacel was used to purify SS-mPEG-GPx1M. Buffer A was 10 mM Tris-HCl (pH 9.0), and buffer B was 10 mM Tris-HCl with 0.1 M NaCl. Briefly, the column materials were equilibrated with buffer A, and then loaded the sample at 0.6 mL/min. Buffer A was used again to obtain a stable baseline, then SS-mPEG-GPx1M was eluted with buffer B at the same flow rate.

### SDS-PAGE

SDS-PAGE separations were performed using a method reported in the literature [39]. Samples were diluted with loading buffer and 20.0 μg of sample was placed into each well. The gels were run at 80 V for 2.5 h. The gels were stained with Coomassie Blue R-250 stain and destained with destaining solution four times.

### Iodine staining procedure

The samples were diluted with loading buffer and 20.0 μg of sample was placed into each well. The gels were run at 80 V for 2.5 h. The gels were then immersed in 2.5% glutaraldehyde solution for fixation. First, the fixed running gel was transferred to 0.1 M perchloric acid (15 mL) for 15 min. Second, 0.1 M iodine solution (2 mL) and 5% barium chloride solution (5 mL) were added using the method by Manfred et al. [40]. The stained gel was transferred into a plastic box and washed with 20 mL of deionized water three times. The iodine-stained PEG bands appeared within a few minutes and were photographed under a camera.

### MALDI-TOF-MS

Reaction mixture and purified SS-mPEG-GPx1M samples were dialyzed 5 times with 500 mL of deionized water each time at 4°C. Sinapinic acid was applied as the matrix. The samples (0.6 μL) were mixed with a 70% aqueous acetonitrile solution (0.6 μL) and then dried at room temperature. Then, the samples were analyzed with a MALDI-TOF (Bruker Autoflex) mass spectrometer [41].

### Enzyme activity assay

The enzyme activities of GPx1M and SS-mPEG-GPx1M were determined according to a method in a previous study [42]. The samples and 50 mM PBS (pH 7.4), 1 mM EDTA, 2 mM GSH, 0.2 mM NADPH, and 1 unit GSH were incubated at 37°C for 5 min. Then, 60 μM hydrogen peroxide (final concentration), as the substrate, was added to start the reaction. The reaction was monitored using an ultraviolet (UV) spectrophotometer at a wavelength of 340 nm. The enzymatic activity was expressed as 1 μM of NADPH oxidized one minute.

### Effect of temperature, pH, and storage time on GPx1M and SS-mPEG-GPx1M activity

#### Determination of GPx1M and SS-mPEG-GPx1M pH activity profiles

Samples (5 μL) of SS-mPEG-GPx1M and GPx1M were each diluted 20-fold with 50 mM PBS with pH values varying from 5.0 to 11.0 and detected for enzymatic activity as described in section “Enzyme activity assay”. All values were converted to a relative value, and the highest enzyme activity was set as 100%.

#### Determination of GPx1M and SS-mPEG-GPx1M temperature activity profiles

Temperature activity studies were applied to GPx1M and SS-mPEG-GPx1M samples in 50 mM PBS (40% glycerin, pH 7.4) as described in section “Enzyme activity assay”. GPx1M and SS-mPEG-GPx1M preparations were incubated at 19, 30, 37, 45, 50 and 55°C for 5 min and assayed for their enzymatic activity as previously described. All values were converted to a relative value, and the highest enzyme activity was set as 100%.

#### Generating storage temperature stability profiles

Storage temperature stability studies of GPx1M and SS-mPEG-GPx1M preparations were also performed. GPx1M and SS-mPEG-GPx1M samples were obtained from 50 mM PBS buffer (40% glycerin, pH 7.4). All samples were prepared at the same protein concentration before storage. GPx1M and SS-mPEG-GPx1M were stored at 4 and −20°C. Their activities were detected over time at indicated time points. The original enzyme activity (0 day) was set as 100%.

#### Effect of trypsin on GPx1M and SS-mPEG-GPx1M activity

The effect of PEGylation on the resistance of GPx1M to protease hydrolysis was evaluated by measuring the enzyme activity of GPx1M and SS-mPEG-GPx1M treated with trypsin for different times. GPx1M and SS-mPEG-GPx1M samples were digested with 10% (w/w) trypsin at 37°C, respectively. Then, the trifluoroacetate (TFA) was used to arrest the digestion at indicated time points. The original enzyme activity (0 min) was set as 100%.

### Determination of PEGylation site

The LC-ESI-MS was used to analyze the PEGylation site. Briefly, after digestion using trypsin at 37°C, peptides in GPx1M and SS-mPEG-GPx1M were extracted with solution consisting of 5% TFA (v/v), 50% acetonitrile (ACN), and 45% ddH_2_O. The RP-HPLC Acclaim PepMap C_18_ column (150 μm i.d. × 150 mm), Ultimate 3000 system (Thermo Fisher Scientific, USA), and Q Exactive™. Hybrid Quadrupole-Orbitrap™. mass spectrometer (Thermo Fisher Scientific, USA) were used to separate, collect, and analyze all digested products of GPx1M and SS-mPEG-GPx1M. The raw MS files were analyzed and searched against target protein database based on the species of the samples using MaxQuant (1.6.2.10).

### Pharmacokinetics analysis of SS-mPEG-GPx1M

All Sprague-Dawley **(**SD) rats were randomly divided into the GPx1M group and the SS-mPEG-GPx1M group with 4 rats in each group after housing on the animal facility for 7 days. A single intraperitoneal injection (i.p.) of GPx1M (100 μg/kg) were given into rats of the GPx1M group. Rats of the SS-mPEG-GPx1M group received SS-mPEG-GPx1M (GPx1M equivalent) with the same way as the GPx1M group. Blood samples at different time points (0, 1, 2, 4, 8, 10, 12, 25, 30, 36, 48, 60, 72, 96, and 120 h) were taken from retroorbital plexus and centrifuged to obtain serum. The concentration of GPx1M or SS-mPEG-GPx1M in serum was assayed by a competitive inhibition ELISA kit. The pharmacokinetic data were calculated with PKSolver 2.0 software [43–45].

### *In vitro* antioxidant effects of SS-mPEG-GPx1M

#### In vitro cell viability

The *in vitro* cell viability was examined in H9c2 cells using the MTT assay [46]. Briefly, H9c2 cells with an initial density of 1 × 10^5^ viable cells/well were plated in 96-well plates and incubated for 48 h at 37°C with 5% CO_2_. The H9c2 cells were then incubated with GPx1M/SS-mPEG-GPx1M (0.01–0.4 U/mL) and ADR (0.5–3 μM), respectively. After 24 h of incubation, 20 μL of MTT solution (5 mg/mL) was added to each well in the plate. After 4 h incubation, the culture medium was extracted, 150 μL of DMSO was placed in each well, and the absorbance was determined at 492 nm using a microplate reader (Epoch, Biotek Instruments, USA). The cell viability was calculated by the following Equation [46]:


$$\text{Cell viability \%}=({\text{A}}_{\text{sample}}-{\text{A}}_{\text{blank}})/({\text{A}}_{\text{control}}-{\text{A}}_{\text{blank}})\times 100\%$$

where A_sample_ represents the values obtained from the samples treated with GPx1M, SS-mPEG-GPx1M, and ADR; A_control_ represents the values obtained from the cells incubated with DMEM medium and A_blank_ represents the values obtained from DMEM medium.

#### Cytotoxicity of pre- and coincubation of GPx1M/SS-mPEG-GPx1M with ADR

To investigate the cytotoxicity during the pre- and coincubation of GPx1M/SS-mPEG-GPx1M with ADR, H9c2 cells with an initial density of 1 × 10^5^ viable cells/well were plated in 96-well plates and incubated for 48 h at 37°C with 5% CO_2_. The H9c2 cells were preincubated with GPx1M/SS-mPEG-GPx1M (0.01–0.4 U/mL) for 1 h and then incubated with an additional 2.5 μM ADR (final concentration) for 24 h or treated with 2.5 μM ADR for 12 h and then incubated with additional GPx1M/SS-mPEG-GPx1M (0.01–0.4 U/mL) for another 12 h. The cell viability was determined using the MTT assay as described previously.

#### Hoechst 33258 assay for apoptosis

H9c2 cells with an initial density of 1 × 10^6^ viable cells/well were plated in 6-well plates and incubated for 48 h at 37°C with 5% CO_2_. The H9c2 cells were preincubated with GPx1M/SS-mPEG-GPx1M (0.08 U/mL) for 1 h and then incubated with additional ADR (2.5 μM) for 24 h or treated with ADR (2.5 μM) for 12 h and then incubated with additional GPx1M/SS-mPEG-GPx1M (0.08 U/mL) for 12 h. Following the incubation period, the cells were fixed in 1 mL of 0.1% formaldehyde for 20 min and then washed with PBS three times. Then, 1 mL of Hoechst 33258 staining reagent (10 μg/mL) was applied to stain the apoptotic cells in the dark at 37°C for 30 min, followed by rinsing with PBS three times. The apoptotic cells were observed and quickly photographed under a Nikon fluorescence microscope at an excitation wavelength of 330–380 nm [47].

#### Reactive oxygen species

The reactive oxygen species (ROS) were detected using an ROS assay kit after 24 h incubation as described in section “Hoechst 33258 assay for apoptosis”. Briefly, the cells were treated with the fluorescent probe 2′, 7′-dichlorofluorescin-diacetate (DCFH-DA) [48, 49]. DCFH-DA was oxidized by ROS and then converted to the fluorescent product 2′, 7′-dichlorofluorescein (DCF). One milliliter of DCFH-DA reagent (10 μM) was added to 6-well plates. After incubation with DCFH-DA for 30 min at 37°C, the cells were rinsed with PBS three times. Then, the cells were quickly observed and photographed under a Nikon fluorescence microscope at an excitation wavelength of 480–535 nm. The fluorescence intensity of ROS was also detected with a Bio-Tek fluorescence microplate reader.

#### Determination of MDA

After a 24 h incubation as described in section “Hoechst 33258 assay for apoptosis”, the culture medium was then discarded. The cells were rinsed with PBS three times and harvested. Then, the cells were rinsed twice more with PBS, resuspended in 100 μL PBS and then ultrasonicated for 10 s. The lipid peroxidation product MDA was detected by means of an MDA assay kit as described in the manufacturer’s instructions. The MDA absorbance values were measured by ultraviolet spectroscopy at 532 nm.

#### Assay of LDH

H9c2 cells were treated as described in the determination of MDA. The intracellular LDH content was evaluated using an LDH assay kit as described in the manufacturer’s instructions. The LDH absorbance value was measured using a microplate reader at 450 nm.

### *In vivo* antioxidant effects of SS-mPEG-GPx1M

The antioxidant study of SS-mPEG-GPx1M was performed in male SD rats (200 ± 20 g). After a week of adaptive feeding, all rats were randomly grouped into the control group (Control), the ADR group (ADR), the GPx1M group (GPx1M), and the SS-mPEG-GPx1M group (SS-mPEG-GPx1M). There were five rats in each group. Rats of the control group and the ADR group were treated with saline (i.p.) for 5 days, and the ADR group was given ADR (15 mg/kg, i.p.) on the 3th day. Rats of the GPx1M group received 4 μg/kg/d GPx1M (i.p.) for 5 days, and the same dose of SS-mPEG-GPx1M (GPx1M equivalent) was used to the SS-mPEG-GPx1M group for 5 days (i.p.). On the 3th day, ADR (15 mg/kg, i.p.) was injected into rats of the GPx1M group and the SS-mPEG-GPx1M group. Blood samples were collected from the rats anesthetized with 5% chloral hydrate at the 6th day, and centrifuged to obtain serum. The rats were sacrificed, and the heart tissues were preserved for H&E staining and DHE staining. The LDH, CK, and MDA of serum were measured according to the manufacturer’s protocol. Heart weight/body weight ratios (HW/BW) of all rats were calculated.

### Statistical analysis

All values are suggested as means ± standard deviations (SD). The Student’s *t*-test was used to analysis the differences between different groups. *p* < 0.05 was considered statistically significant.

### Highlights

GPx1M was modified for the first time by SS-mPEG (*M*_w_ = 5 kDa). SS-mPEG-GPx1M showed better stability to thermal and pH changes than GPx1M. GPx1M and SS-mPEG-GPx1M displayed similar antioxidant activity *in vitro*. SS-mPEG-GPx1M had a better antioxidant effect than GPx1M *in vivo*.
